# Cargo-specific effects of hypoxia on clathrin-mediated trafficking

**DOI:** 10.1007/s00424-024-02911-6

**Published:** 2024-01-31

**Authors:** Gijsbert J. van Belle, Anke Zieseniss, Doris Heidenreich, Maxime Olmos, Asia Zhuikova, Wiebke Möbius, Maarten W. Paul, Dörthe M. Katschinski

**Affiliations:** 1 Institute of Cardiovascular Physiology, University Medical Center Göttingen, Georg-August University, 37073 Göttingen, Germany; 2 Department of Neurogenetics, Electron Microscopy, City Campus, Max-Planck-Institute for Multidisciplinary Sciences, 37075 Göttingen, Germany; 3 Department of Molecular Genetics, Oncode Institute, Erasmus MC Cancer Institute, Erasmus University Medical Center, Rotterdam, Netherlands

**Keywords:** Hypoxia, Endocytosis, Clathrin, Live cell microscopy

## Abstract

Clathrin-associated trafficking is a major mechanism for intracellular communication, as well as for cells to communicate with the extracellular environment. A decreased oxygen availability termed hypoxia has been described to influence this mechanism in the past. Mostly biochemical studies were applied in these analyses, which miss spatiotemporal information. We have applied live cell microscopy and a newly developed analysis script in combination with a GFP-tagged clathrin-expressing cell line to obtain insight into the dynamics of the effect of hypoxia. Number, mobility and directionality of clathrin-coated vesicles were analysed in non-stimulated cells as well as after stimulation with epidermal growth factor (EGF) or transferrin in normoxic and hypoxic conditions. These data reveal cargo-specific effects, which would not be observable with biochemical methods or with fixed cells and add to the understanding of cell physiology in hypoxia. The stimulus-dependent consequences were also reflected in the final cellular output, i.e. decreased EGF signaling and in contrast increased iron uptake in hypoxia.

**Supplementary Information:**

The online version contains supplementary material available at 10.1007/s00424-024-02911-6.

## Introduction

Communication with the extracellular space is vital for the preservation of cellular homeostasis. Endocytosis provides in this need by engulfing external materials with the cell membrane and absorbing it for further processing. Clathrin-mediated endocytosis (CME) is one of the best studied pathways that plays an important role in endocytosis as a whole [6]. CME is involved in the uptake of extracellular fluid and receptor-bound ligands [5, 28, 35]. Two important examples of possible ligands are the epidermal growth factor (EGF) and ferrous iron in complex with the glycoprotein transferrin (TF) [12]. These ligands bind their respective specific receptors, after which receptor activation and CME mediated internalization can occur. Aside from CME, clathrin is also involved in trans-Golgi-derived vesicle trafficking, which transports proteins from the trans-Golgi network (TGN) to various destinations within the cell or for secretion. The dysregulation of endocytosis or intracellular trafficking can provide cancer cells with an increase in proliferative and metastatic potential [10]. This dysregulation can arise when components of the endocytic machinery are mutated, have altered expression patterns or when changes in protein turnover occur [21].

Cancer cells often have to deal with low oxygen environments due to their uncontrolled growth [14]. Such hypoxic tumours in most entities are characterized by severe disease progression and a poor prognosis. Both the deregulation of signalling via the EGF receptor and the increase in iron uptake have been described as driven by hypoxia [23, 39]. On a more global scale, it has been shown that hypoxia downregulates the total membrane proteome through a decelerated protein internalization [4]. Interestingly, some surface proteins like the carbonic anhydrase IX, integrin α-2 and the C-X-C chemokine receptor type 4 are able to override this effect, which adds to their enhanced cell membrane surface abundance aside from increased expression in hypoxia. For the receptor tyrosine kinase EGFR and transferrin receptors (TFR), on the other hand, a delayed recycling, which results in a slowed deactivation of the receptors, has been described [39]. In these studies, long-term hypoxia models were applied either through incubation of the cells at 1% O_2_ for 20 h [4] or by using cell lines with a loss of function of the von Hippel-Lindau protein [39]. In consequence, these cells stabilize the hypoxia-inducible factor (HIF)α and show a chronic activation of the respective target genes. None of the hypoxia-related studies analysed spatiotemporal dynamics of clathrin-mediated transport. To obtain this information, we used live cell microscopy in normoxia and hypoxia with and without ligand stimulation of cells in the present study. Microscopy approaches are well suitable for analysing intracellular trafficking [8]. Live cell techniques, in particular, can provide insights into the dynamics of both vesicles and their cargo. Most research investigating dynamics concerning clathrin and cargo mobility has employed live cell total internal reflection fluorescence (TIRF) microscopy [1, 22, 25, 37], albeit with occasionally conflicting results. This technique offers a high signal-to-noise ratio, rapid imaging speed and reduced photobleaching [27]. However, it has a limitation — it only functions in proximity to the plasma membrane and has a penetration depth of ~ 100 nm into the cell, missing information about dynamic events further towards the centroid of the cells. To address this limitation, we utilized epifluorescence live cell microscopy under normoxic and hypoxic conditions. We employed newly developed GFP-tagged clathrin-expressing cells and a self-made vesicle tracking/quantification plugin. These cells were analysed under non-stimulated conditions or after stimulation with fluorescently labelled EGF or transferrin.

## Results

### Hypoxia decreases the number of clathrin vesicles and their localization

**Fig. 1:**
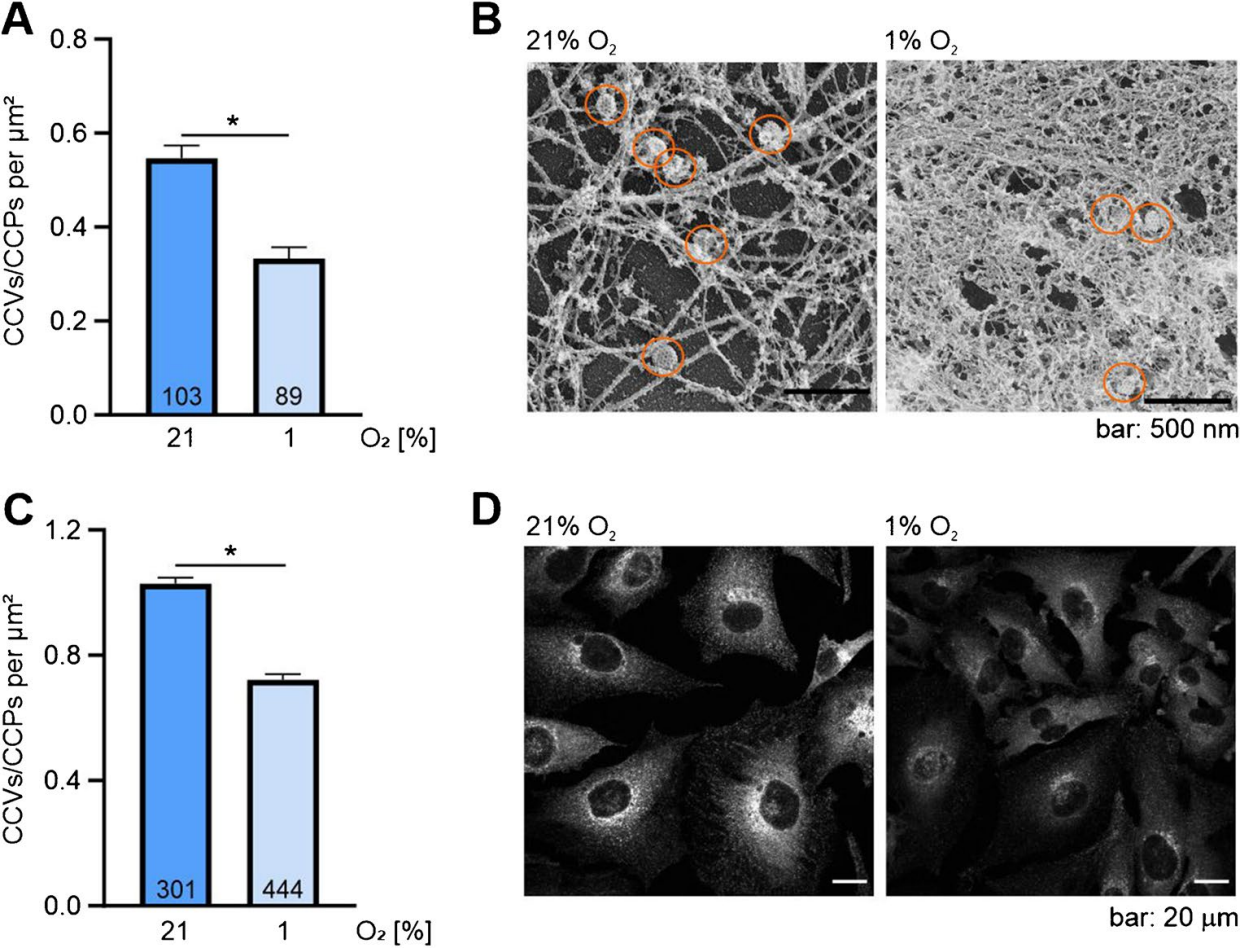
The number of clathrin-coated vesicles/particles is reduced in hypoxia. **A** MDA-MB-231 cells were subjected to either 6 h of normoxia (21% O_2_) or hypoxia (1% O_2_) before fixation, electron microscopy and subsequent quantification of clathrin-coated vesicles/particles. In total, 103 and 89 cells incubated in normoxia or hypoxia were included in the analysis. **B** Representative EM micrographs of the experiment analysed in **A**. **C** MDA-MB-231 cells were subjected to 6 h of normoxia (21% O_2_) or hypoxia (1% O_2_) before fixation, immunofluorescence staining for clathrin and subsequent quantification. In total, 301 and 444 cells incubated in normoxia or hypoxia were included in the analysis. **D** Representative immunofluorescence micrographs of the experiment analysed in **C**. Mean ± SEM, unpaired *t*-test with Welch’s correction, *p < 0.05

To analyse if hypoxia affects clathrin-associated trafficking in unstimulated cells, we quantified clathrin vesicles after we exposed MDA-MB 231 breast cancer cells to 6 h of hypoxia (1% O_2_) compared to normoxia (21% O_2_). To this end, we used electron microscopy and clathrin immunofluorescence stainings. Both approaches provide information about the overall abundance of clathrin-associated vesicles; however, they are not able to distinguish between clathrin vesicles (CCVs) derived from endocytosis or the TGN and clathrin-coated particles that are still associated to the plasma membrane. The TGN-associated vesicles were indeed visible by an intense clathrin staining in regions associated with the Golgi apparatus. Overall, we found a decrease in the number of clathrin-associated vesicles/particles in hypoxic samples quantified using electron microscopy (Fig. 1A and B) and clathrin immunofluorescence staining (Fig. 1C and D). Please note that we also observed a significant increase in actin filament density in hypoxia, which we have described earlier [41].

**Fig. 2:**
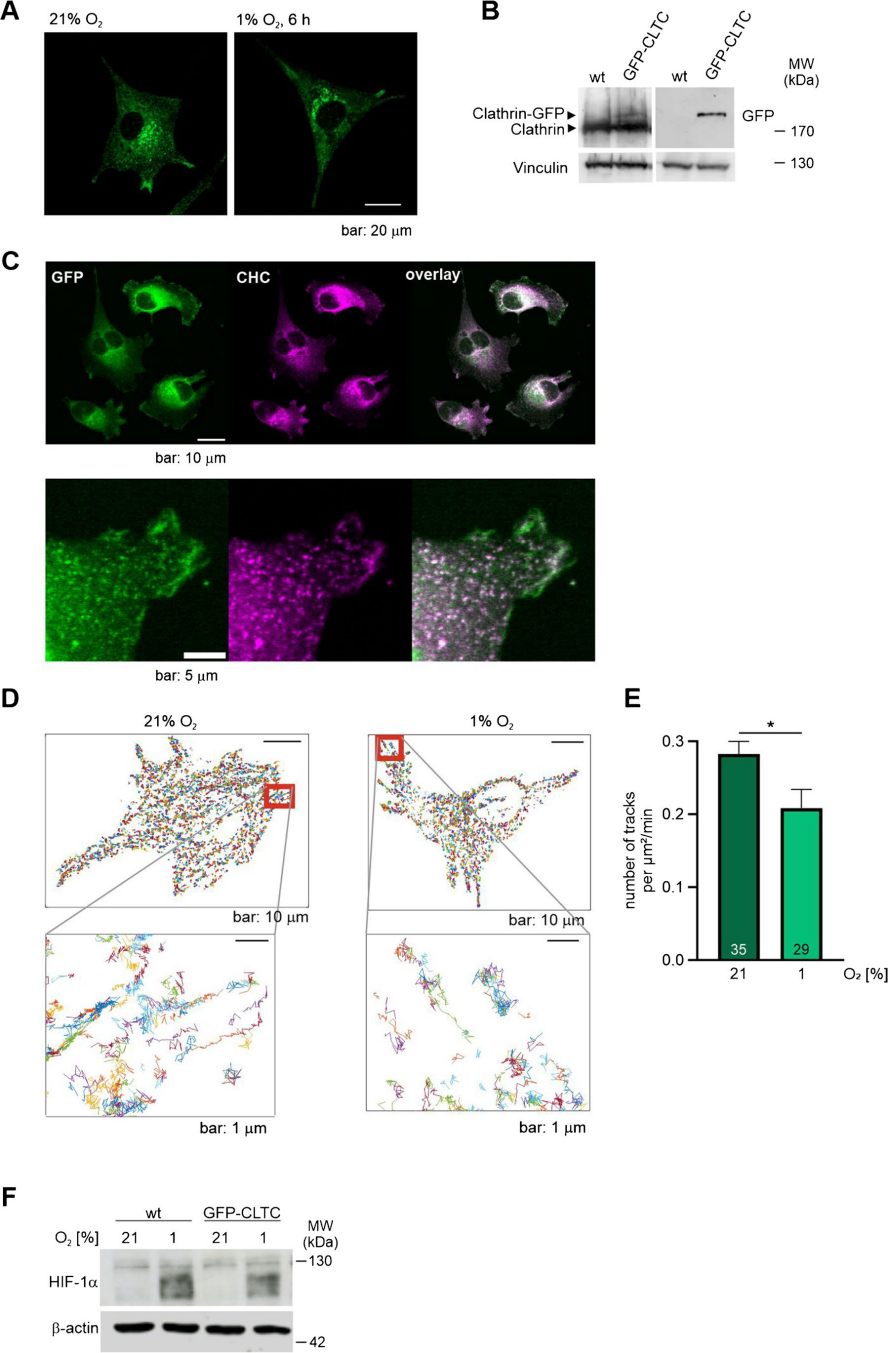
Analysis of GFP-tagged clathrin reporter MDA-MB-231 cells confirm a reduced number of clathrin-coated vesicles in hypoxia. **A** Representative micrograph of a fixed MDA-MB-231 cell stably expressing GFP-clathrin after incubation in normoxia (21% O_2_) or hypoxia (1% O_2_) for 6 h. **B** MDA-MB-231 wild type (wt) and GFP-CLTC expressing MDA-MB-231 (GFP-CLTC) cells were analysed for clathrin and GFP protein levels by immunoblotting. Vinculin was used as a loading control. **C** Immunofluorescence staining of fixed MDA-MB-231 cells stably expressing GFP-clathrin with an anti-clathrin heavy chain (CHC) antibody. **D** Representative images with plotted accumulated tracks of MDA-MB-231 GFP-CTLC cells which were incubated for either 6 h in normoxia (21% O_2_) or hypoxia (1% O_2_). The image sequences were analysed using the SOS plugin for ImageJ, and tracks were generated. Area in the red box is enlarged as an example. **E** Quantification of number of tracks in MDA-MB-231 GFP-CLTC from the images obtained in **D**. 35 and 29 cells were analysed in normoxia and hypoxia. **F** HIF-1α immunoblot in MDA-MB-231 wt and GFP-CLTL cells after incubating the cells in normoxia or hypoxia for 6 h. β-actin was used as a loading control. Mean ± SEM, unpaired *t*-test with Welch’s correction, *p < 0.05

To further investigate the spatial–temporal changes in the vesicle behaviour, we generated a clathrin reporter MDA-MB 231 cell line that stably expresses GFP-CLTC (Fig. 2A and B). Co-staining of GFP-CLTC cells with an anti-clathrin heavy chain antibody demonstrated that the majority of the clathrin vesicles were marked by GFP (Fig. 2C). The cells were used to follow clathrin over time for 6 h in hypoxia or normoxia using live cell time-lapse imaging. In the recorded time series, single clathrin-associated vesicles were tracked using the SOS plugin for ImageJ [30]. We could confirm a significant decrease in the number of tracked vesicles in hypoxia in these live cells (Fig. 2D and E; supplementary files 1–4). To ensure that with the introduction of the GFP-CLTC in the MDA-MB 231 cells the capability of the cell to adapt to the hypoxic environment is not disturbed, we analysed the HIF-1α protein levels in both wild type and GFP-CLTC expressing cells. In both samples, we detected a similar stabilization of the HIF-1α protein (Fig. 2F).

### Hypoxia changes intracellular trafficking

**Fig. 3:**
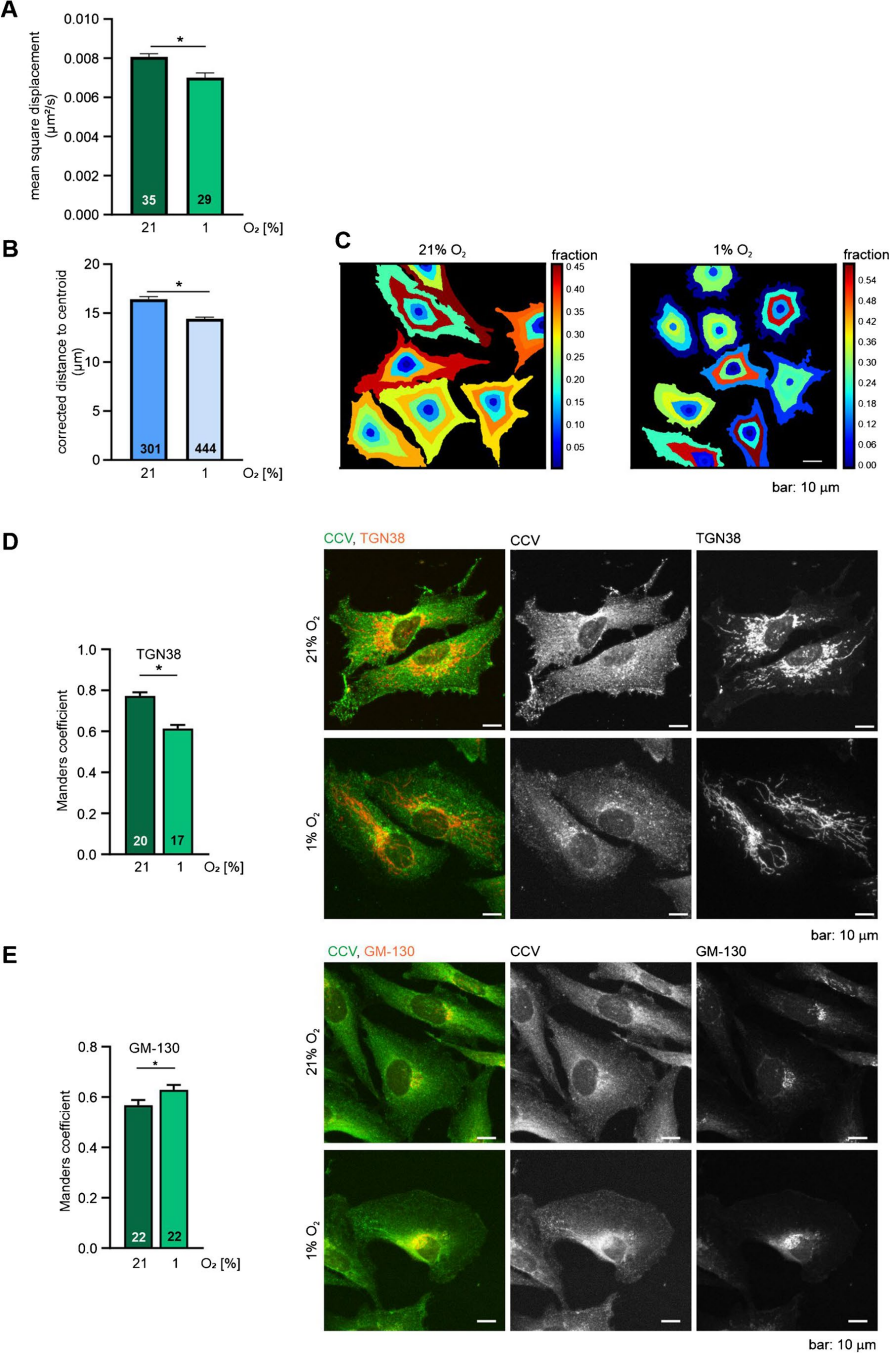
Hypoxia alters the mobility and cellular localization of intracellular vesicles. Quantification of mean square displacement (**A**) and corrected distance to centroid (**B**) of GFP-tagged vesicles measured in MDA-MB-231 GFP-CLTC cells which were imaged either in normoxia (21% O_2_) or hypoxia (1% O_2_) for 6 h. **C** Exemplary pseudo-coloured images showing the fraction at distance of MD-MB-231 GFP CLTC cells obtained from the experiment performed in **B**. **D**, **E** Quantification of the Manders overlap coefficient measured in fixed MDA-MB-231 cells which were subjected to either normoxia or hypoxia for 6 h followed by immunofluorescence co-staining for CTLC and TGN38 (**D**) or GM-130 (**E**). Representative stainings are shown on the right side. Numbers in the bars indicate the number of the cells analysed per condition. Mean ± SEM, unpaired *t*-test with Welch’s correction, *p < 0.05

The mobility of particles can be characterized using the mean square displacement (MSD), which measures how a particle’s position deviates from a reference point over time. We utilized the tracks generated from live cell time-lapse imaging to calculate the MSD of the tagged vesicles (see supplementary files 1–4). These tracks were imported into MATLAB and analysed using a custom script. The resulting MSD values were found to be comparable to MSDs reported for clathrin-coated structures quantified by TIRF microscopy [29]. When comparing vesicle mobility in normoxia, and after 6 h of hypoxia, we detected a significantly lower MSD in the hypoxic samples (Fig. 3A). To see if this change in mobility is reflected in the localization of the vesicles within the cell, we quantified the mean distance to the centroid of single vesicles in our immunofluorescence stained samples using the CellProfiler software [19]. GFP-marked vesicles were found more towards the centroid of the cells, which were incubated for 6 h in hypoxia, after cell area correction (Fig. 3B and C). Since endocytic trafficking via clathrin is also known to be directed towards the Golgi apparatus and clathrin also mediates the transport from the TGN [15, 32], we looked for differences in the colocalization of clathrin with two different Golgi markers: TGN38, a marker for the trans-Golgi stack, and GM130, a marker for the cis-Golgi stack. When comparing the Manders overlap coefficient (MOC) of the clathrin signal with the TGN38 signal after 6 h of exposure to 21% O_2_ or 1% O_2_, we detected a significant decrease in the colocalization in the hypoxic condition (Fig. 3D). In the GM130 stained samples, the MOC shows the opposite effect with a significant increase in colocalization with clathrin (Fig. 3E). Taken together, cells that have been subjected to 6 h of hypoxia show less mobile vesicles and the vesicles that are still there are localized more towards the centroid of the cells. This localization fits with the increase in colocalization of clathrin with the cis-Golgi, which is located more towards the centroid of the cell compared to the trans-Golgi.

### Ligand-specific effects of hypoxia on number, mobility and lifetime of cargo-loaded vesicles

**Fig. 4:**
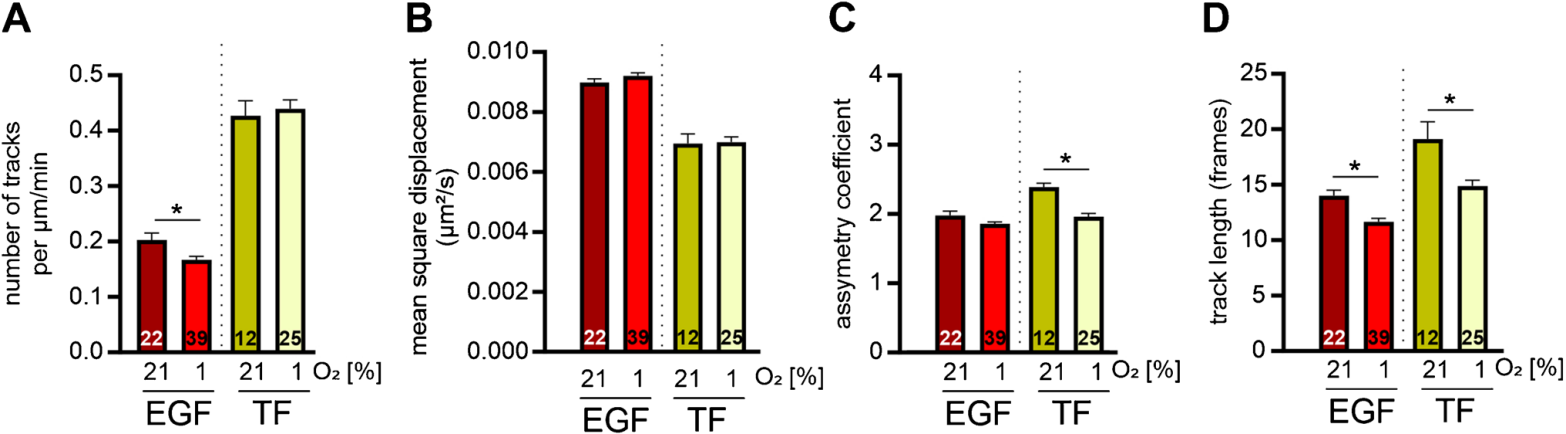
The effects of hypoxia on clathrin-coated vesicles (CCVs) are cargo specific. MDA-MB-231 GFP-CLTC cells were subjected to normoxia (21% O_2_) or hypoxia (1% O_2_) for 80 min. Cells were stimulated with EGF or transferrin (TF). CCVs and cargo were imaged in their respective fluorescent channel and tracks were generated accordingly. Tracks were quantified regarding number (**A**), mean square displacement (**B**), asymmetry coefficient (**C**) and track length (**D**). Numbers in the bars indicate the number of cells analysed. Mean ± SEM, unpaired *t*-test with Welch’s correction, *p < 0.05

Having established the reporter cell line and the analysis software, we wanted to investigate how hypoxia would influence vesicles loaded with different cargos in a next step. To this end, we stimulated the MDA-MB-231 GFP-CLTC cells with either fluorescently tagged EGF or TF. Since EGF and TF are mainly endocytosed via CME, mostly CCVs derived from the plasma membrane were quantified in these experiments. The stimulation was performed at 4 °C to ensure a complete stop of internalization of bound cargo, and the cells were only returned to 37 °C at the start of imaging. After release, the cells were followed for 80 min in either hypoxia or normoxia. Subsequently, the EGF- and TF-loaded CCVs were analysed. As readout, we determined the number of tracks (Fig. 4A; supplemental files 5–8) and the MSD (Fig. 4B) after the different stimulations. Overall, we observed a lower number of tracks for the EGF-loaded vesicles compared to the TF stimulation. The mobility of the EGF-loaded CCVs, however, was higher compared to the TF loaded CCVs reflected by the MSD. Hypoxia further decreased the number of EGF-loaded CCVs, whereas no impact neither on the number of TF-loaded vesicles nor the MSDs for both cargoes was observed. To link the number and mobility of the cargo-loaded CCVs to directed motion of the vesicles, we calculated the asymmetry coefficient (AC) [13, 20]. In brief, we first measured the angle between the directions of CCV displacements for three consecutive time points of the tracks. The AC was then defined as the ratio between the frequencies of forward (180 ± 30°) versus backward (± 30°) movements per cell. The lower the AC, the more directionality. In EGF-stimulated cells, the EGF-loaded CCVs did not show a different AC after 80 min when comparing normoxia to hypoxia (Fig. 4C), whereas in sharp contrast, TF-loaded CCVs showed a higher directionality in hypoxia. Finally, we characterized the cargo-loaded CCVs by track length (Fig. 4D). Track length was analysed as number of frames, in which the vesicle can be observed. It is a metric that reports about the turnover and lifetime of the CCVs. In the EGF as well as TF stimulation experiments, we observed a significantly shorter track length in hypoxia indicating a shorter lifetime of the cargo loaded CCVs. Taken together, whereas this shorter lifetime was associated with a decreased number of CCVs after EGF-stimulation, TF-loaded CCVs had a higher directionality.

### Differential effect of hypoxia on EGF and TF signaling

**Fig. 5:**
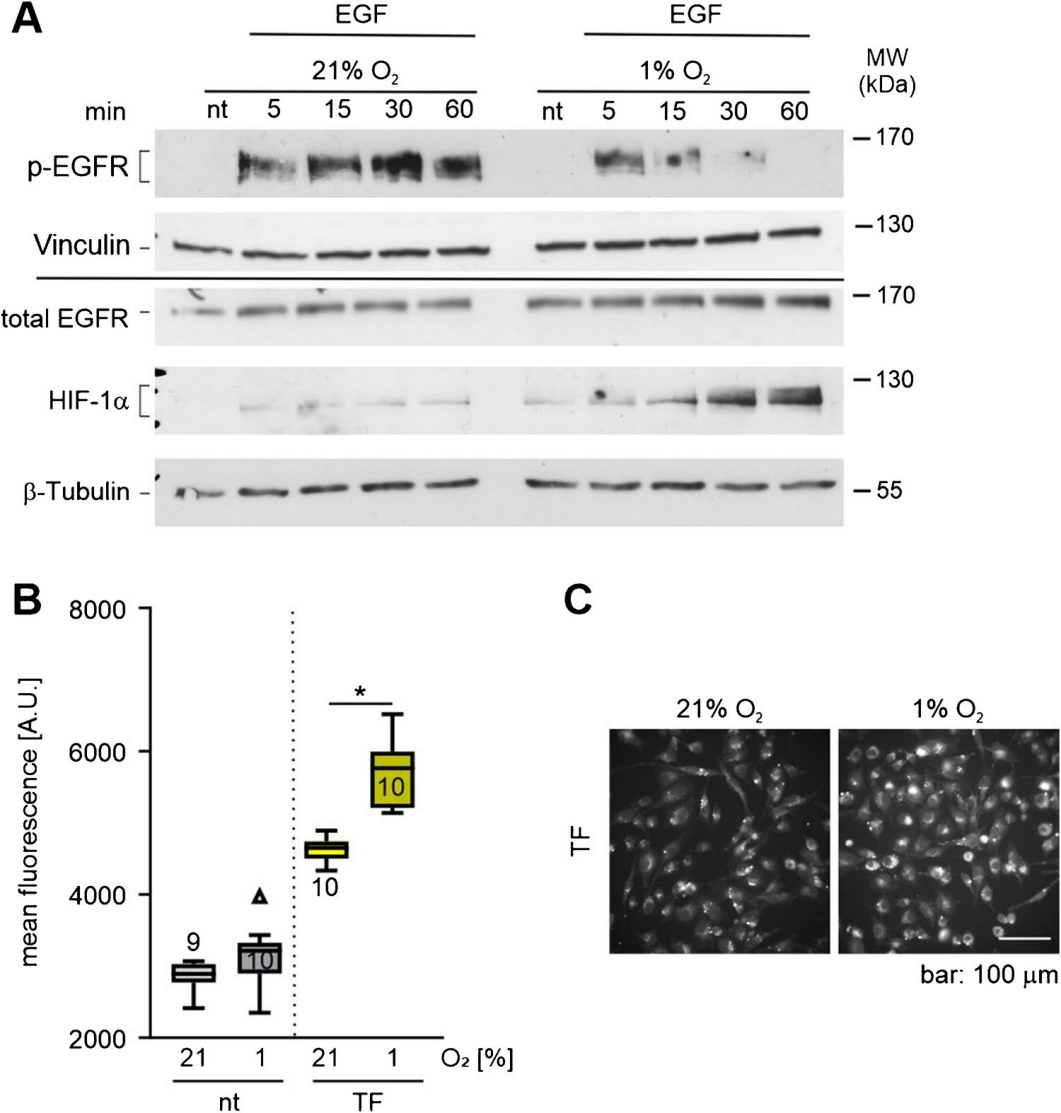
Hypoxia affects the final cellular output differently after EGF and transferrin (TF) stimulation. **A** Immunoblots showing total HIF-1α, EGFR and phosho-EGFR protein level in normoxia (21% O_2_) and hypoxia (1% O_2_) for the indicated time points in MDA-MB-231 cells after stimulation with EGF. Vinculin and β-tubulin served as loading controls. **B** Quantification of intracellular Fe^2+^ in MDA-MB-231 cells using FerroOrange after stimulation with TF in normoxia or hypoxia for 80 min. Numbers in the bars indicate number of image sections analysed; box and whisker plot shows median and range of values. **C** Representative micrographs from the experiment in **B**. Unpaired *t*-test with Welch’s correction, *p < 0.05

To assess if the differential effect of hypoxia on EGF versus TF-loaded CCVs is reflected in altered signalling of the two stimuli, we analysed in a next step EGF receptor (EGFR) phosphorylation and iron uptake. The binding of EGF to the EGFR triggers persistent phosphorylation of said receptors [17]. To see if hypoxia affects the duration of this phosphorylation, MDA-MB-231 cells were stimulated with EGF similarly to the live cell experiments and samples were prepared at different time points. Protein levels of phospo-EGFR and total EGFR of these samples were checked via Western blotting (Fig. 5A). We detected stable activation and phosphorylation of the EGFR in normoxia persisting until at least 60 min after stimulation. Although EGFR showed a likewise robust phosphorylation in hypoxia, the phosphorylation was already decreasing 15 min after the stimulation and was almost absent after 60 min. This finding is in line with the lower number of EGF-loaded CCVs in hypoxia.

To assess the effect of hypoxia on MDA-MB-231 cells on iron uptake after stimulation, we used the FerroOrange fluorescent probe. This probe enables live cell imaging of intracellular Fe^2+^ (Fig. 5B and C). An increase in fluorescent intensity in cells treated with TF compared to non-treated cells was clearly visible. Iron uptake was further enhanced when TF stimulation was performed in hypoxia. This gain of function in TF downstream effect is of clear difference to the suppressive effect of hypoxia on the EGFR signalling.

## Discussion

The effect of hypoxia on intracellular trafficking has been studied previously with in part contradictory results as to whether endocytic uptake and intracellular trafficking are increased or decreased in hypoxia [26]. Mostly, biochemical methods and microscopy techniques with fixed cells were applied in these hypoxia-related studies. Live cell imaging has gained importance for understanding cellular functions [2]. It allows to obtain spatiotemporal information of subcellular events in real time. CME is a complex and dynamic cellular event, which should be analysed close to native conditions to prevent perturbations by the experimental settings [18, 31]. In line with this, live cell fluorescence microscopy has also contributed to the understanding of protein dynamics and localization at endocytic sites [3, 7, 37]. To tease apart the effects of hypoxia on clathrin dependent events, we applied live cell imaging to our research question. This undertaking was supported by the development of a GFP-tagged clathrin-expressing cell line. We used this cell line without stimulation with EGF or transferrin to obtain information on all clathrin-dependent trafficking processes including CCVs deriving from CME or from intracellular trafficking from the TGN. CME is initiated by the recruitment of adaptor proteins that link clathrin to the underlying membrane via their association with charged plasma membrane lipids and couple the assembly of the clathrin coat with the selection of transmembrane cargo proteins [9]. Thus, in the EGF and transferrin stimulation experiments, we obtained insight into CME events. Additionally, we utilized a newly developed plugin analysis tool to assess the properties of the tagged vesicles, including their number, mobility, directionality and lifetime. The newly developed SOS plugin was specifically tailored for analysing data obtained through epifluorescence microscopy. A previously established CME analysis program was found to be less compatible with non-TIRF microscopy data and was slower in processing the large imaging datasets obtained in our study [38]. Using our newly developed tool set, we demonstrate that the effects of hypoxia differ depending on the cargo that is endocytosed. Whereas EGF-loaded CCVs were reduced in number as well as in lifetime in hypoxia, TF-loaded CCVs gained directionality. Changes in the endocytosis of select membrane proteins could be used to switch on or to amplify specific pathways counteracting changes in the environment by the cell. In line with the decreased number of EGF-loaded CCVs, we observed a shortened EGFR phosphorylation in hypoxia. On the other hand, the uptake of iron in consequence of TF stimulation was enhanced. Iron is essential for oxygen transport and is a component of molecular O_2_-carrying proteins, such as haemoglobin and myoglobin [16]. Iron is also a constituent of redox enzymes and iron-sulfur clusters. Therefore, the balance between the control of iron availability and the physiology of hypoxic responses is critical for maintaining cell homeostasis. The increased directionality of TF-loaded CCVs and alongside increased iron uptake thus is in line with the intricate link between iron homeostasis and oxygen availability [34]. Contradictory results have been reported for the effect of hypoxia on EGFR signalling in the past. Evidences have shown that hypoxic microenvironment in solid tumours not only translationally upregulates EGFR expression through HIF-2α [11], but also transactivates EGFR and triggers its internalization and non-degradative endosomal accumulation [36]. This arrest of EGFR at non-degradative endosomes in hypoxia has been mostly observed in cell lines with a constitutive high activation of the HIF system due to a defective pVHL-mediated proteasomal degradation. The endosomal arrest mimics to some extent a stress response, which results in a similar phenomenon [36]. Cell context–dependent, however, a hypoxia-mediated EGFR downregulation has been reported likewise including experiments with the poorly differentiated triple-negative breast cancer cell line MDA-MB-231 that was also used in the present study [24]. Our data indicate that EGF-loaded CCVs are specifically affected by acute hypoxia, which results in a decreased EGFR signalling. A direct comparison to the effects after TF stimulation reveals that the effects of hypoxia are cargo-dependent with impact on the final cellular output.

Taken together, we applied live-time imaging together with a clathrin reporter cell line and a newly developed software plugin, which allowed us to track the abundance of CCVs and characterize the CME events after stimulating cells with EGF or TF in normoxia and hypoxia. These data reveal cargo-specific effects, which would not be observable with biochemical methods or fixed cells.

## Material and methods

### Cell lines and cell culture

MDA-MB 231 cells were obtained from the *Deutsche Sammlung von Mikroorganismen und Zellkultur* (DSMZ). Cells were cultured in high glucose DMEM (PAN-Biotech) supplemented with 10% heat inactivated FCS (PAN-Biotech) and 1% penicillin/streptomycin (P/S) (PAN-Biotech). Experiments were performed in a regular humidified incubator in normoxic conditions at 37 °C, 5% CO_2_ and 21% O_2_ (Binder) or in hypoxic conditions (37 °C, 5% CO_2_, 1% O_2_) using an adjustable hypoxic humidified workstation (invivo300; Ruskin Technology). Cells were tested for myocoplasma contamination on a regular basis by PCR.

### Generation of MDA-MB 231 GFP-CLTC cells

The GFP-CHC17KDP plasmid was a gift from Stephen Royle and obtained from Addgene (plasmid # 59799; http://n2t.net/addgene:59799; RRID:Addgene_59799). MDA-MB 231 cells were transfected using Lipofectamin (Invitrogen) according to the manufacturer’s protocol. After 48 h, the selection with G418 (Gibco) was started. Cells were kept under antibiotic selection for 2 weeks to ensure integration and stable expression. The expression of GFP-CHC17KDP was checked using fluorescent microscopy and Western blot.

### Electron microscopy

Electron microscopy was performed similarly as previously described [40]. MDA-MB-231 cells were grown on 8-mm glass coverslips (Th. Geyer) and incubated in normoxic (21% O_2_) or hypoxic (1% O_2_) conditions for the specified time. Before experimentation, the cells were washed with pre-warmed PBS. To visualize labile and easily extractable components, a concurrent extraction/light fixation was performed using 0.5% Triton-X100 and 0.25% glutaraldehyde (EM grade, Science Services, Munich, Germany) for 7 min. Further fixation was performed in three steps: (1) 20 min incubation with 2% glutaraldehyde in HEM buffer (100 mM HEPES, 1 mM EGTA, 1 mM MgCl_2_; pH 6.9); (2) 20 min incubation with 0.1% tannic acid, followed by three careful washes with water and 5 min incubation in water; and (3) 20 min incubation with 0.1% uranyl acetate (SPI Chemicals, West Chester PA, USA) in water. Samples were dehydrated by 10 min incubations in aqueous solution with stepwise increasing ethanol concentrations (30%, 50%, 70%, 90% and 100%). For contrasting, the samples were incubated for 20 min in 0.1–0.2% uranyl acetate in 100% ethanol. Samples were transferred to a critical point dryer (Leica EM CPD300) for further dehydration overnight. Before critical point drying, the samples were first exposed to 10 times exchanges with liquid CO_2_ at 5–10 °C. Dried samples were sputter coated using the ACE600 (Leica Microsystems, Vienna, Austria) with a ~ 2.7-nm-thick Pt layer during rotation at an angle of 45° and a layer of 3–5 nm carbon at an angle of 90°. The Pt/C replicas were detached from the coverslip by floating on 10% hydrofluoric acid. Replicas were then mounted on formvar-coated 100 mesh hexagonal copper TEM grids (Science Services, Munich, Germany). EM pictures were taken with the LEO 912 AB electron microscope (Carl Zeiss Microscopy, Oberkochen, Germany) using an on-axis 2k CCD camera (TRS, Moorenweis, Germany).

### Immunofluorescence staining

MDA-MB 231 cells were seeded on 24-mm coverslips (Th. Geyer) and incubated in normoxic (21% O_2_) or hypoxic (1% O_2_) conditions for the specified time. Cells were fixed using 4% paraformaldehyde for 20 min under normoxic or hypoxic conditions. Fixed cells were then washed three times with PBS, permeabilized for 5 min in 100 µM digitonin in PBS and washed again three times with PBS. Cells were blocked by incubating cells with 2% BSA in PBS for 20 min and then washed three times with PBS. Cells were incubated with primary antibody (anti-clathrin heavy chain, Abcam, Ab21679, 1:500; anti-GM-130, BD Biosciences, 610822, 1:100; anti-TGN-38, BD Biosciences, 610899, 1:500) for 2 h at room temperature, washed three times with PBS and then incubated with secondary antibody (anti-rabbit-FITC, Santa Cruz, Sc-2012, 1:100; anti-mouse-Rhodamine, BD Biosciences, 610822, 1:100, anti-rabbit rhodamine Sc-2091) for 1 h at room temperature. This was followed by washing three times with PBS. Finally, cells were counterstained with DAPI (1:500) for 5 min. Coverslips were then mounted on glass slides using Flouromount (Sigma, SLBR5174V) aqueous mounting medium.

### Fluorescently labeled cargo stimulation and live cell imaging

For live cell imaging, MDA-MB-231 GFP-CLTC cells were placed under normoxic (21% O_2_) or hypoxic (1% O_2_) conditions on ice for 5 min to halt trafficking processes. Medium was replaced with either 2 µg/mL tetramethylrhodamine-conjugated EGF (Invitrogen, E3481) or 25 µg/mL Alexa Fluor 546 conjugated TF (Invitrogen, T23364), diluted into a HEPES based buffer with pH 7.4 supplemented with 20 mM glucose and 1% BSA for 5 min with the cells still being on ice. Finally, this solution was replaced with FluoroBrite DMEM (Gibco) supplemented with 5% FCS (PAN-Biotech) and GlutaMAX 100x (200 mM) (Gibco). The cells were then transported on ice and, if needed in an airtight box to retain hypoxia, to the microscope.

For intracellular iron ion measurements, MDA-MB-231 cells were seeded as for live cell imaging. The cells were stimulated as per the live cell imaging stimulation protocol. After 80 min, the medium was removed; the cells were washed three times with HBSS, and they were incubated with 1 µmol/L FerroOrange (Dojindo, F374-12) in HBSS for 30 min before imaging.

Imaging was performed on an inverted Olympus IX83 widefield microscope equipped with an ORCA-flash4.0 digital camera (C11440, Hamamatsu Photonics, Japan) and a MT20 illumination system (Olympus) as light source and a 60 × UPLS Apo/1.35 objective (Olympus). The acquisition of images was performed via the CellSens software with either a 20 × objective (UPLSAPO20X, NA: 0.75, Olympus) or a 60 × objective (UPLSAPO60X, NA: 1.35, Olympus). Experiments were performed with the CellVivo incubation setup (Pecon) in normoxic conditions at 37 °C, 5% CO_2_ and 21% O_2_ or in hypoxic conditions at 37 °C, 5% CO_2_ and 1% O_2_. Live cell images were recorded with a 500-ms exposure time and a total 1 s interval between images for the 80-min stimulation experiments. For the FerroOrange experiments, 500 ms exposure time was used without intervals.

### Quantification of colocalization

Z-stack micrographs from stained samples were obtained using a confocal laser scanning microscope LSM 510 Meta (Carl Zeiss, Jena, Germany) equipped with a 63 × plan-apochromatic phase-contrast water-immersion objective, NA 1.4. Fluorophores were excited either with light at 488 nm from an argon laser or at 543 nm from a HeNe laser. The emission of the fluorophores was recorded using the Meta detector. For measuring the Mander overlap coefficient, the *Colocalisation Threshold* function of ImageJ [33] was used. The channels of the obtained Z-stacks of the recorded micrographs were split and background subtracted using a rolling ball radius of 50 pixels and no smoothing. The channel combination was set to red:green and ROI2 as a mask.

### Quantification of distance to centroid

Z-stack micrographs from stained samples were obtained using a confocal laser scanning microscope LSM 510 Meta (Carl Zeiss, Jena, Germany) equipped with a 63 × plan-apochromatic phase-contrast water-immersion objective, NA 1.4. Fluorophores were excited either with light at 488 nm from an argon laser. Using ImageJ, the Z-stacks were flattened into maximum intensity Z projections and saved. From these maximum projection files, mask files were generated. This was done by performing a background subtraction with a rolling ball radius of 50 pixels. The intensity was set with a minimum of 3 and a maximum of 30, and afterwards, blurring with a Gaussian blur with a sigma radius of 5 was applied. The original Z projections were loaded into CellProfiler 2.2.0 [19] together with their respective masks. An inhouse composed CellProfiler pipeline was used for further analysis. Cell segmentation was performed by running the IdentifyPrimaryObjects module to identify single cells in the mask images. An automatic, per object two class Otsu thresholding strategy was followed. Cells touching the boarder were discarded. For better detection of CCVs in the original image, the EnhanceorSuppressFeatures module was performed on the slow/circular setting, enhancing Speckles with a feature size of 20. This enhanced image was then masked with the previously generated segmented mask using the MaskImage module, and in this newly masked enhanced image, the enhanced CCVs were identified using the IdentifyPrimaryObject Module using a global background thresholding method. Clumped objects were distinguished using the intensity setting. The MeasureObjectIntensity, MeasureImageAreaOccupied, MeasureImageIntenisty and MeasureObjectSizeShape modules were used to measure the intensity and area of different cells and the enhanced CCVs. Using the ExpandOrShrinkObjects module, the enhanced identified CCVs were shrank to points to remove the intensity variable by using the ConvertObjectsToImage in binary mode. These were then related to their parent cell using the RelateObjects module, and the child-parent centroid distance was calculated. Using the MeasureObjectIntensityDistribution module, the fraction at distance of these centroid distance was plotted. Finally, all data was exported to a csv file using the ExportToSpreadsheet module. Since we noticed a change in cell area in hypoxic conditions, we corrected for this bias. The correction was performed by first letting the CellProfiler software calculate the mean distance to centroid. The square root of the area of the hypoxic cell was divided by this mean distance of this cell to get a normalization factor (alpha); alpha = sqrt(area hypoxic cell)/(mean distance to centroid hypoxic cell). The mean distance to the centroid was multiplied with this normalization factor to get a value which was comparable to the normoxic sample.

### Quantification of FerroOrange staining in live cells

ImageJ was used for quantification of the mean intensity of FerroOrange stained cells. Images were thresholded to only select the area covered by cells. Then, the mean fluorescence of the selected area was measured. Three experiments were performed with a minimum of nine images for each condition. One representative experiment is shown.

### Vesicle tracking and quantification

Tracking was performed using the SOS plugin for ImageJ, which is available from http://www.smal.ws/home/software. The plugin first applied a spot detection algorithm based on a weighted least-squares Gaussian fitting of PSFs, accepting localizations above a threshold of 5 a.u. above background intensity and a sigma value between 1 and 15 pixels. Tracks were subsequently created by linking the detections using a nearest-neighbour-linking approach, where subsequent localization within a 3-pixel radius was taken into account. Furthermore, a cutoff value of a minimum of five frames was used and a maximum gap size of five frames between subsequent frames. The tracking data was visualized using the MTrackJ plugin for ImageJ.

The data obtained with the SOS-plugin was further processed with a MATLAB script. For individual tracked vesicles, the mean-square-displacement was calculated as

$$MSD\left(\tau \right)=\frac{1}{N-\tau }\sum_{t=1}^{N-\tau }{({x}_{t+\tau }-{x}_{t})}^{2}+{({y}_{t+\tau }-{y}_{t})}^{2}, \tau =1,\dots ,N-1$$

Subsequently, the apparent diffusion constants were derived from the first four time steps (*τ*) of the slope of the *MSD*(*τ*) curve as

$$MSD(t)=4Dt$$

For the estimation of the asymmetry coefficient, the angle (*α*) between subsequent displacement was calculated as

$$\alpha =\left|{{\text{tan}}}^{-1}\left(\frac{{\text{det}}\left[\left(\begin{array}{cc}{v}_{1}& {v}_{2}\end{array}\right)\right]}{{\text{dot}}\left({v}_{1},{v}_{2}\right)}\right)\right| ,\mathrm{ where}\;v1={\left({{p}_{2}-p}_{1}\right)}^{\prime}\; {\text{and}}\; v2=\left({{p}_{3}-p}_{2}\right)^{\prime}$$

The asymmetry coefficient was defined as the fraction of displacements with an angle equal or above 15° divided over the fraction of displacements with an angle equal or smaller than 30°.

The scripts for calculating the apparent diffusion and the asymmetry coefficient can be found at https://github.com/maartenpaul/vesicle-tracking-visualization

### Western blots

Cells were lysed with 400 mM NaCl, 1 mM EDTA, 10 mM Tris–HCl pH 8 and 0.1% Triton X-100 supplemented with protease inhibitors (Roche) or urea buffer (3.7 M Urea, 135 mM Tris/HCL pH 6.8, 1% SDS, 1% Igepal (NP-40)). Protein concentrations were quantified using the DC protein assay (Bio-Rad). For immunoblot analysis, protein samples were resolved by SDS-PAGE and transferred onto nitrocellulose membranes (Amersham Biosciences, Freiburg, Germany) by semidry blotting (PeqLab, Erlangen, Germany). Primary antibodies: anti-HIF-1α (#610822, Transduction Lab, Heidelberg, Germany), anti-clathrin heavy chain (Abcam, ab21679), anti-vinculin (V9264, Sigma), anti-β-actin (Sigma, A 5441), anti-β-tubulin (Abcam, ab6046), anti-GFP (#2555, CST), anti-phospho-EGFR (Y1173) (Abcam, 53A5), anti-EGFR (CST, 4267).

Secondary antibodies: anti-rabbit IgG HRP (Millipore, AP187p), anti-mouse-HRP IgG/IgM (Millipore, AP130P). Membranes were incubated with chemiluminescent HRP substrate (Millipore, Darmstadt, Germany).

### Statistical analysis

Statistical analyses for the experiments were performed using GraphPad Prism software 9.0.0 (GraphPad Software). Specific tests, *p* values and sample size can be found in the figures and the figure legends.

### Supplementary Information

Below is the link to the electronic supplementary material.

Supplementary file 1. Representative assembled figures of a MDA-MB-231 cell stably expressing GFP-CLTC (green). The file shows a timespan of 120 seconds with each figure representing an interval of 1 second where the cell is incubated in 21% O_2_. Left panel: original image, middle panel: marked detections, right panel: filtered detections. Scale bar represents 10 *µ*m. (TIF 295 MB)

Supplementary file 2. Representative assembled figures from a zoom in of a MDA-MB-231 cell stably expressing GFP-CLTC (green). The file shows a timespan of 120 seconds with each figure representing an interval of 1 second where the cell is incubated in 21% O_2_. Left panel: original image, middle panel: marked detections, right panel: filtered detections. Scale bar represents 5 *µ*m. (TIF 666 MB)

Supplementary file 3. Representative assembled figures of a MDA-MB-231 cell stably expressing GFP-CLTC (green). The file shows a timespan of 120 seconds with each figure representing an interval of 1 second where the cell is incubated in 1% O_2_. Left panel: original image, middle panel: marked detections, right panel: filtered detections. Scale bar represents 10 *µ*m. (TIF 584 MB)

Supplementary file 4. Representative assembled figures from a zoom in of a MDA-MB-231 cell stably expressing GFP-CLTC (green). The file shows a timespan of 120 seconds with each figure representing an interval of 1 second where the cell is incubated in 1% O_2_. Left panel: original image, middle panel: marked detections, right panel: filtered detections. Scale bar represents 5 *µ*m. (TIF 588 MB)

Supplementary file 5. Representative movie of a MDA-MB-231 cell stably expressing GFP-CLTC (green) stimulated with EGF (red). Overlap of the signals is represented in yellow. The movie shows a timespan of 120 seconds of a movie with an interval of 1 second where the cell is incubated in 21% O_2_. The movie is played back at 25 FPS. Scale bar represents 10 *µ*m. (AVI 31.8 MB)

Supplementary file 6. Representative movie of a MDA-MB-231 cell stably expressing GFP-CLTC (green) stimulated with EGF (red). Overlap of the signals is represented in yellow. The movie shows a timespan of 120 seconds of a movie with an interval of 1 second where the cell is incubated in 1% O_2_. The movie is played back at 25 FPS. Scale bar represents 10 *µ*m. (AVI 16.0 MB)

Supplementary file 7. Representative movie of a MDA-MB-231 cell stably expressing GFP-CLTC (green) stimulated with transferrin (red). Overlap of the signals is represented in yellow. The movie shows a timespan of 120 seconds of a movie with an interval of 1 second where the cell is incubated in 21% O_2_. The movie is played back at 25 FPS. Scale bar represents 10 *µ*m. (AVI 13.4 MB)

Supplementary file 8. Representative movie of a MDA-MB-231 cell stably expressing GFP-CLTC (green) stimulated with transferrin (red). Overlap of the signals is represented in yellow. The movie shows a timespan of 120 seconds of a movie with an interval of 1 second where the cell is incubated in 1% O_2_. The movie is played back at 25 FPS. Scale bar represents 10 *µ*m. (AVI 35.2 MB)

## Data Availability

The data and material that support the findings of this study are available from the corresponding author upon request. The described scripts for calculating the apparent diffusion and the asymmetry coefficient are freely accessible and can be found at https://github.com/maartenpaul/vesicle-tracking-visualization.
